# Novel archaeal thermostable cellulases from an oil reservoir metagenome

**DOI:** 10.1186/s13568-017-0485-z

**Published:** 2017-09-29

**Authors:** Anna Lewin, Jinglie Zhou, Vu Thuy Trang Pham, Tone Haugen, Mohamed El Zeiny, Olav Aarstad, Wolfgang Liebl, Alexander Wentzel, Mark R. Liles

**Affiliations:** 1 Department of Biotechnology and Nanomedicine, SINTEF Materials and Chemistry, Richard Birkelands vei 3B, 7465 Trondheim, Norway; 2 Department of Biological Sciences, Auburn University, 101 Life Sciences Building, 120W, Samford Avenue, Auburn, AL 36849 USA; 3 Department of Microbiology, Technical University of Munich, Freising-Weihenstephan, Germany; 4 Norwegian University of Science and Technology (NTNU), Trondheim, Norway

**Keywords:** Oil reservoir, Cellulase, Cellulose degradation, CAZymes, Enzyme discovery, Functional metagenomics

## Abstract

Microbial assemblages were sampled from an offshore deep sub-surface petroleum reservoir 2.5 km below the ocean floor off the coast of Norway, providing conditions of high temperature and pressure, to identify new thermostable enzymes. In this study, we used DNA sequences obtained directly from the sample metagenome and from a derived fosmid library to survey the functional diversity of this extreme habitat. The metagenomic fosmid library containing 11,520 clones was screened using function- and sequence-based methods to identify recombinant clones expressing carbohydrate-degrading enzymes. Open reading frames (ORFs) encoding carbohydrate-degrading enzymes were predicted by BLAST against the CAZy database, and many fosmid clones expressing carbohydrate-degrading activities were discovered by functional screening using *Escherichia coli* as a heterologous host. Each complete ORF predicted to encode a cellulase identified from sequence- or function-based screening was subcloned in an expression vector. Five subclones was found to have significant activity using a fluorescent cellulose model substrate, and three of these were observed to be highly thermostable. Based on phylogenetic analyses, the thermostable cellulases were derived from thermophilic *Archaea* and are distinct from known cellulases. Cellulase F1, obtained from function-based screening, contains two distinct cellulase modules, perhaps resulting from fusion of two archaeal cellulases and with a novel protein structure that may result in enhanced activity and thermostability. This enzyme was found to exhibit exocellulase function and to have a remarkably high activity compared to commercially available enzymes. Results from this study highlight the complementarity of hybrid approaches for enzyme discovery, combining sequence- and function-based screening.

**Electronic supplementary material:**

The online version of this article (doi:10.1186/s13568-017-0485-z) contains supplementary material, which is available to authorized users.

## Introduction

Extremophiles have attracted great interest for their ability to thrive in conditions that sometimes include multiple environmental extremes and for their enzymes that have adapted to these extreme conditions and therefore may have industrial applications (Rothschild and Mancinelli 2001). *Taq* DNA polymerase is the classic example of an enzyme from a thermophile, i.e. *Thermus aquaticus*, isolated in culture from hot springs at Yellowstone National Park (Chien et al. 1976). The traditional approach used to identify and exploit these enzymes is culture-dependent, which greatly restricts the accessible diversity of thermophile-derived natural products.

The relatively small percentage of microorganisms that can be readily cultured under laboratory conditions has led to the development of novel culture methods and the adoption of culture-independent methods (Amann et al. 1995; Ferrer et al. 2007; Lewin et al. 2013). However, the application of novel cultivation strategies is slow and will not enable cultivation of the extant diversity of microbial genomes from extreme environments, necessitating new approaches to exploit natural products from as-yet-uncultured microbes for industrial application (Daniel 2004). The use of a culture-independent metagenomic approach permits access to microbial genomes and their biologically active molecules through isolation of DNA from environmental microbes followed by direct sequencing or cloning DNA to generate a metagenomic library (Handelsman 2004; Banik and Brady 2010). The library can then be screened by both sequence-based and function-based methods for natural product discovery (Delmont et al. 2011; Milshteyn et al. 2014). Each of these approaches has its own set of biases, including the potential inability to heterologously express a cloned gene due to differences in the transcriptional and/or translational apparatus of the host, and biases associated with annotation of shotgun sequences. Even with these well-recognized methodological biases, a metagenomic approach has been previously shown to be effective in discovery of enzymes with novel activities (Wilson and Piel 2013; Milshteyn et al. 2014). In fact, the very first published example of a functional metagenomic approach for enzyme discovery involved the cloning of cellulases from “zoolibraries” (Healy et al. 1995). Recently, metagenomic approaches have been used to discover many novel carbohydrate-active enzymes (CAZymes) from soil (Jiang et al. 2009; Nacke et al. 2012), cow rumens (Pope et al. 2010), sediments (Klippel et al. 2014), biochemical reactors (Mewis et al. 2013) and aquatic environments (Rebuffet et al. 2011; Martin et al. 2014).

Petroleum reservoirs contain complex hydrocarbons trapped in porous rock formations and contain varying amounts of formation water that is high in salt content. In two previous studies, samples were collected from a reservoir 2.5 km below the sea floor of the Norwegian Sea with an in situ temperature of 85 °C and pressure of 250 bars (Kotlar et al. 2011; Lewin et al. 2014). The oil-producing wells used for sampling never experienced any injection of microorganisms from the surface or other habitats, and during sampling, contamination and cell lysis due to rapid shifts in temperature and pressure were minimized by using a pressurized chamber to allow a slow and controlled reduction of temperature and pressure (Kotlar et al. 2011; Lewin et al. 2014). Oil reservoirs like the sampled habitat provide multiple extreme conditions, including high pressure, temperature and salt, though they contain abundant carbon sources that in principle could support microbial growth (Magot et al. 2000). Microorganisms have adapted to such extreme conditions through the evolution of e.g. thermostable enzymes (Rothschild and Mancinelli 2001).

The present study focused on the discovery of thermostable CAZymes, particularly cellulases, from an oil reservoir metagenome using both sequence-based and function-based screening. Cellulases are glycoside hydrolases (GH) that are able to hydrolyze 1,4-β-d-glycosidic linkages in cellulose, hemicellulose, lichenin, and cereal β-d-glucans. The CAZy database currently lists a total of 131 GH families, 17 of which include enzymes with cellulase activities (Lombard et al. 2013). Cellulases have many applications, including biofuel production (e.g. cellulosic ethanol) by decomposition of plant-derived cellulose into monosaccharides that can be used in fermentation (Brethauer and Wyman 2010). However, biofuel production processes such as simultaneous saccharification and fermentation (SSF) or separated hydrolysis and fermentation (SHF) can profit from high temperature, which would inhibit the activity of many currently available cellulolytic enzymes (Lynd et al. 2002). Therefore, identification of novel thermostable cellulases could be a vital step in improving the efficiency and economy of cellulosic biofuel production. The availability of specific carbon substrates (e.g., cellulose) within these oil reservoir samples was unknown, but given the history of formation of these petroleum reservoirs essentially from ancient plant biomass plankton, it has been hypothesized that carbohydrate-degrading enzymes would be encoded in the metagenomes of microorganisms populating such a habitat and would have evolved enhanced thermal stability (Tissot and Welte 1978).

## Materials and methods

### Oil reservoir sampling, DNA isolation and handling

Oil reservoir samples were collected and metagenomic DNA isolated as described in two previous studies (Kotlar et al. 2011; Lewin et al. 2014). Isolated DNA was used in direct 454 pyrosequencing for metagenomic analysis of the phylogenetic and functional diversity of oil reservoir microbial assemblages (Kotlar et al. 2011; Lewin et al. 2014). For preparation of a fosmid library, DNA was amplified using Phi29 polymerase (WGA; Qiagen REPL midi kit, Qiagen Sciences, Germantown, USA) in individual reactions of 50 µl and pooled after amplification. Two individual rounds of amplification were conducted using DNA from each sample (water and oil phases). The amplified DNA was isolated and purified using the Qiagen QIAamp DNA mini kit according to the manufacturer’s protocol and used in fosmid library construction using the Epicentre pCC1FOS system (Epicentre, Madison, WI, USA), as described in (Aakvik et al. 2009) and (Xu et al. 2014). The 11,520 clones of the resulting fosmid library were arrayed in 30 × 384-well microtiter plates.

### Extraction of fosmid DNA from the fosmid library

The fosmid library was cultivated in 384-well format at 37 °C on LB medium supplemented with 12.5 µg/ml chloramphenicol and 0.01% (v/w) arabinose. Clones from each two of the 30,384-well plates were pooled, followed by fosmid DNAs extraction using the Qiagen Large-Construct Kit and incubation with Plasmid-Safe™ ATP-Dependent DNase (Epicentre) to reduce chromosomal contamination.

### High throughput sequencing of the fosmid library

The 15 pooled fosmid DNA preparations were fragmented, uniquely barcoded, and purified using the Nextera DNA Sample Prep Kit (Illumina, San Diego, CA) and the DNA Clean and Concentrator Kit (Zymo Research, Orange, CA). Amplification was then performed according to standard Illumina protocols. The amplified library was purified using a Size-Select IT kit (Omega Biotek, Norcross, GA) and DNA quantified using a Qubit fluorimeter (Life Technologies, Carlsbad, CA, USA). Pooled and denaturated DNA was used for Illumina HiSeq sequencing using (2 × 100 bp paired end sequencing chemistry (Illumina, San Diego, CA). The paired end sequencing data of all 15 fosmid DNA pools, as well as the 454 pyrosequencing datasets of previous studies (Kotlar et al. 2011; Lewin et al. 2014) are accessible at http://tare.medisin.ntnu.no/data/mg-oil-compare/.

### Bioinformatics analysis of the fosmid library and shotgun sequences

Two different strategies were used for sequence-based screening. In the first strategy, only Illumina HiSeq reads from the fosmid library were used for assembly and further sequence-based screening. The raw sequences generated by HiSeq were imported into the CLC Genomics Workbench (Qiagen, Cambridge, MA), and trimmed at a stringency of 0.01 (equivalent to Q score of > 40). Trimmed sequences were assembled de novo using the CLC Genomics Workbench to generate a set of contigs per each fosmid pool. Open Reading Frame (ORF) prediction was then performed using “ORF finder by six-reading-frame” on Camera Portal 2.0 (Sun et al. 2010). The predicted ORFs were used for a batch BLASTp against the CAZy database using the tool dbCAN for identification of carbohydrate-degrading enzymes, as well as lipases/esterases (Yin et al. 2012; Lombard et al. 2013). In addition, all raw sequence reads recovered from the fosmid library were also exported to MG-RAST (Project No. mgp7584, access via http://tare.medisin.ntnu.no/data/mg-oil-compare/) to profile microbial diversity and abundance based on phylogeny and function (Meyer et al. 2008). In order to compare microbial diversity present within the fosmid library (plates 3, 4, 9, 10, 13, 14, 17 and 18) to that of 454 pyrosequencing reads from the same sample (Well II), trimmed sequence reads from direct 454 pyrosequencing (Kotlar et al. 2011; Lewin et al. 2014) were also uploaded to MG-RAST for analysis. Organism abundances were predicted using “Best hit classification” (Max. e-Value Cutoff: 1e-5, Annotation Sources: M5NR). Functional abundances were predicted using “hierarchical classification” (Max. e-Value Cutoff: 1e-5, Annotation Sources: Subsystems).

In the second strategy, the fosmid library HiSeq reads from plates 3, 4, 9, 10, 13, 14, 17, and 18 were combined with sequences generated from direct 454 pyrosequencing from the same sample (Well II) in order to achieve longer contigs. The raw 454 sequence reads from direct sequencing were imported into the CLC Genomics Workbench and trimmed at a stringency of 0.01 (equivalent to Q score of > 40). Subsampling of trimmed direct pyrosequencing reads was performed, and the best de novo assembly statistics were obtained from the use of 90% of the reads. The resulting contigs were then used for assembly with reads from Illumina HiSeq sequencing of the fosmid library. The HiSeq reads from the selected fosmid library plates were also subsampled and separately assembled de novo with contigs resulting from the 90% sub-sampling of the reads using SPAdes 3.5.0 (Bankevich et al. 2012). The best assembly was generated using 20% of the sub-sampled Illumina fosmid library reads together with contigs from the direct 454 pyrosequencing. The contigs resulting from each of the above assembly strategies were used for ORF prediction using Prodigal (Hyatt et al. 2010). The predicted ORFs were used for a batch BLASTp against the CAZy database using the tool dbCAN for identification of CAZymes as well as lipases/esterases (Yin et al. 2012; Lombard et al. 2013).

### Functional screening of the fosmid library for carbohydrate-degrading activity

Assays for five different hydrolase enzymatic assays were conducted with five substrates to functionally screen the fosmid library. In each assay, the *Escherichia coli* fosmid clones, pre-grown overnight (96-well plates, 200 μl LB + chloramphenicol (12.5 μg/ml) per well, 37 °C, 200 rpm), were inoculated onto the respective agar medium (with 0.01% arabinose). Cellulase and xylanase activities were screened using LB agar containing 0.1% carboxymethylcellulose (CMC) and 0.1% xylan (beech wood), respectively (Kasana et al. 2008; Krishnan et al. 2012). Amylase assay was done on starch (Peltier and Beckord 1945), the protease assay utilized 2% skim milk (Sokol et al. 1979) and LB agar with 1% tributyrin was used to detect the activity of esterases/lipases (Ertugrul et al. 2007). After 37 °C incubation overnight, all agar plates, except starch agar plates, were incubated at 60 °C overnight and further fumigated with chloroform for 1 h to lyse *E. coli* cells. Halos of clones expressing proteases or esterases/lipases could be directly observed. For the three other enzymatic assays, colonies were first removed using 95% ethanol and dH_2_O. CMC and xylan agar plates were stained using 1% Congo red (15 min, de-stained using 3 M NaCl). For starch agar plates, cell lysis was achieved by fumigation (chloroform, 1 h, room temperature), followed by iodine staining (0.3% iodine and 0.6% potassium iodine, 15 min). The positive clones were re-streaked from original wells onto agar plates with their respective substrates, and tested for validation. Only clones that were validated as positive upon re-testing were selected for further analyses.

### Sequencing fosmid clones that express cellulase activity

Fosmid clones with reproducible cellulase activity were selected for next-generation sequencing. Cultivated fosmid clones (LB + 12.5 μg/ml chloramphenicol + 0.01% arabinose, 37 °C overnight) were subjected to individual fosmid DNA extraction using the Large-Construct DNA isolation kit (Qiagen). Extracted fosmid DNA was processed with the Nextera DNA Sample Prep Kit (Illumina, San Diego, CA) and sequenced using Illumina MiSeq with 2 × 300 bp paired-end chemistry (Illumina, San Diego, CA). Obtained sequences were trimmed, assembled de novo, and ORFs were predicted using the CLC Genomics Workbench. Predicted cellulase ORFs from each clone were annotated by a BLASTp search.

### Subcloning of cellulase genes

Predicted cellulase-encoding ORFs from six clones expressing cellulase activity along with complete or nearly complete cellulase gene ORFs identified from pooled library sequencing were selected for subcloning. Each respective ORF was PCR amplified and subcloned into the Expresso Rhamnose SUMO subcloning system (Lucigen, Middleton, WI) and used to transform *‘E. cloni* 10G’ cells (Lucigen) by electroporation. Subclones able to express a cellulase activity were selected after growing on CMC agar and staining (1% Congo red, 15 min).

Genes encoding four cellulase candidates were also synthesized as *E. coli* codon optimized variants (Genscript, Piscataway, NJ, USA), delivered cloned in vector pUC57 (http://www.genscript.com/vector/SD1176-pUC57_plasmid_DNA.html). The codon-optimized genes were subcloned into the pRham N-His SUMO expression vector as described above, which was used for transformation of chemically competent *‘E. cloni* 10G’ cells.

### Thermal stability test of subclones with cellulase activity

Two methods were used to evaluate the thermal stability of cellulases produced by subclones expressing cellulase activity. Culture broth of each clone (0.2% rhamnose and 30 μg/ml kanamycin (Km), 37 °C overnight) was collected and heated at a series of temperatures for different incubation times. In the first method, supernatants of cell lysates (using chloroform) of clones were heated at 37, 60, 70, and 80 °C for 1, 2, 3, or 6 h, then spotted onto CMC agar plate, and those with yellow halos were recorded. The second method utilized 4-methylumbelliferyl-β-d-cellobioside (MUC), a fluorescent cellulase substrate, to quantify cellulase thermal stability (Lehmann et al. 2012). MUC has been widely used to assay exocellulase activity, and has also been reported to be capable of detecting endo-cellulase and β-glucosidase activities (Chernoglazov et al. 1989; Kim et al. 2007; Akcapinar et al. 2011; Chen et al. 2012; Ko et al. 2013). Equal volumes of 100 µM MUC were added to heated supernatants of cell lysates for 6 h in a 96-well plate followed by incubation at 37 °C overnight. Next day, the fluorescence of each well was monitored using an excitation at 375 nm and emission of 445 nm with a BioTek Cytation 3 plate reader (Thermo Fisher Scientific Inc).

### Purification of active cellulases from subclones

For production of cellulase enzymes, the respective production strains along with *E. cloni* 10G as a negative control were cultivated in 1000 mL batches (LB media containing 30 µg/ml kanamycin (except for the neg. control) and 0.05% glucose) with a starting OD_600_ of approx. 0.04. The cultures were cultivated at 37 °C and 225 rpm until OD_600_ ≈ 1.4, when the cells were washed to remove glucose and re-suspended in fresh LB + kanamycin + rhamnose (0.2% final conc.) for induction of gene expression. The culture was incubated further until OD_600_ ≈ 4.5 (approx. 8.5 h), when cell mass was harvested and stored at − 20 °C. Crude cell extracts (CCE) were prepared by sonication in 5–10 ml buffer (50 mM potassium acetate buffer, pH5.5) for 7 min (50% duty cycle and output control 4) followed by centrifugation at 20,000 × *g* for 30 min at 4 °C. Isolated extracts were used in heat stability analysis (extract incubation at 70 °C for 3 h), activity assay (MUC assay as described above, as well as substrate specificity analysis as described below) and for isolation of the enzymes by Ni–NTA affinity chromatography. For enzyme purification, 450 µl of sterile filtered (0.2 µm) cell extracts were incubated with 1 ml Ni–NTA agarose (equilibrated with native binding buffer; 50 mM sodium phosphate buffer (pH8.0) with 0.5 M NaCl, 10 mM imidazole and 1 mM DTT) for 60 min at room temperature (RT) in a Rotamixer. Agarose beads were washed in native wash buffer, re-suspended in 2 ml of the same buffer and applied in a plastic column. The beads were washed three times with 5 ml wash buffer and the bound proteins thereafter eluted using elution buffer [50 mM sodium phosphate buffer pH8.0, with 0.5 M NaCl, and 1 mM dithiothreitol (DTT)] with stepwise increasing concentrations of imidazole (100, 150, 200, 250 and 500 mM) in 1 ml fractions. Isolated proteins were subjected to heat incubation (65 °C for 20 min), and used in a cellulase activity assay.

### Substrate specificity analysis

For analysis of a cellulase candidate substrate specificity, a crude cell extract (CCE) containing enzyme was heated at 65 °C for 20 min followed by centrifugation at 13,000×*g* for 5 min at 4 °C for removal of heat labile host proteins. The remaining heat-treated crude cell extract was kept at 4 °C until assayed on different cellulose substrates [carboxymethylcellulose (CMC; Sigma-Aldrich C5678, Sigma-Aldrich, St Louis, Mi, USA), and microcrystalline cellulose (Avicel; Avicel^®^ PH-101; Sigma 11,363)], either undiluted or 5× diluted, and either alone or together with one or two additional commercial cellulose-acting enzymes. Enzyme reactions were carried out in 96 well plates wherein 150 µl of substrate (stock 10 g/l) were subjected to enzymatic reactions with one or several enzymes, 20 µl of each enzyme (3 x 20 µl for three enzyme reactions, 2 × 20 µl for two enzyme reactions and 1 × 20 µl for one enzyme reactions), with 50 mM potassium acetate buffer pH 5.5 added up to a total of 60 µl per well. The amounts of enzyme added in the volume of 20 µl were for endoglucanase (Sigma E2164) 1.05 mg/ml (2.1 U), cellobiohydrolase (Sigma E6412) 1.95 mg/ml (0.14 U) and beta-glucosidase (Sigma 49,290) 0.55 mg/ml (3.3 U). Enzyme reactions were conducted in triplicate at 50 °C for 60 min, followed by heat inactivation of the enzyme(s) at 90 °C for 15 min and centrifugation (10 min at 5000×*g*). The supernatant after centrifugation was used for analysis of the enzyme reaction products. Glucose as a reaction product was quantified by the Amplex Ultra Red assay (Molecular Probes, Thermo Scientific, Waltham, MA, USA) in 96-well microtiter plate format. 50 µl hydrolysate after enzyme reaction was mixed with 50 µl pre-assembled reaction solution (0.1 mM Amplex Ultra Red, 1 U HRP, 10 U glucose oxidase in phosphate buffer (pH 7.4), total volume 5 ml), and the mixture was incubated in the dark for 15 min, followed by fluorescence reading (excitation/emission at 530/590 nm). Amplex Ultra Red detects H_2_O_2_, which is formed from free glucose by glucose oxidase (Invitrogen, Molecular Probes Amplex UltraRed protocol) in the reaction solution.

In addition, products were detected from enzymatic reaction (50 °C overnight) using as a substrate microcrystalline cellulose (Avicel; Avicel^®^ PH-101; Sigma 11363), cellohexaose (Megazyme Wicklow, Ireland), or borohydride reduced cellohexaose (reduced in NaBH_4_ (4% w/v) after incubation at 23 °C for 3 h). The samples were then cooled on ice, neutralized with 1 M acetic acid to decompose surplus NaBH_4_ and dialyzed (Medicell, Republic of Singapore, MWCO = 1 kDa) against MilliQ water. Other substrates tested at a range of 0.03–5 mg/ml were cellobiose (Megazyme), β-glucan (Megazyme; Mw = 89,000 g/mol, hydrolysed at 80 °C for 16 h in 0.1 M HCl, neutralized with 0.1 M NaOH, dialysed (Medicell, MWCO = 3.5 kDa) and freeze dried), xyloglucan, maltoheptaose and maltohexaose (Sigma-Aldrich), each of which were assayed using ion chromatography (ICS) after incubation with enzyme in 25 mM potassium acetate buffer (pH 5.5) at 50 °C overnight. Reaction mixtures without enzyme, as well as a dilution series of a mixture of d-glucose, cellobiose and cellohexaose (1–50 mg/l) were used as references. ICS analysis (ICS-5000 + system (Thermo Scientific) equipped with a pulsed amperometric detector) was performed by applying the reaction mixture to a 4 × 250 mm CarboPac PA100 column connected with a CarboPac PA-100 (4 × 50) mm guard column using isocratic 100 mM NaOH and a linear gradient from 10 to 410 mM sodium acetate in 60 min at a flow rate of 1 ml/min. Data were collected and processed using Chromeleon 7.2 software (Thermo Scientific). Degradation of chitosan samples (2 ml, 5 mg/ml) was analyzed by injection of chitosan samples incubated with and without enzyme containing heated CCE on three Superdex 30 columns (2.6 cm × 60 cm, serially connected) eluted with 0.15 M ammonium acetate buffer, pH 4.5 at a flow rate of 0.8 ml/min. The eluent was monitored with an online refractive index detector (Shodex RI-101).

## Results

### Functional and phylogenetic classification of direct and fosmid-derived metagenomic sequences

**Fig. 1:**
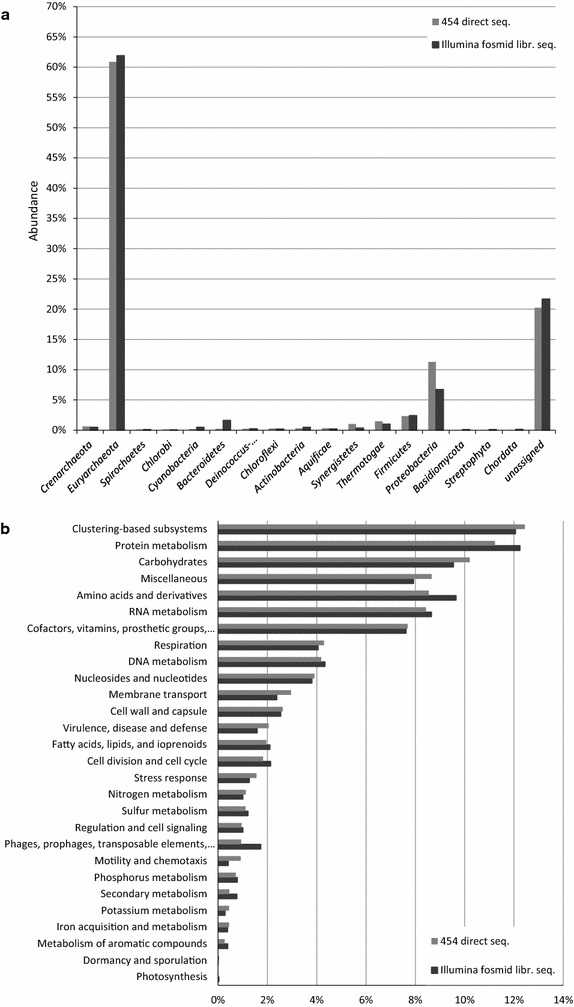
Comparison of direct 454 (light grey) and fosmid library (dark grey) sequencing results. **a** Relative abundance of direct 454 sequences and fosmid metagenomic library Illumina sequences at the phylum level, and **b** based on functional classification as compared to the SEED database (Overbeek et al. 2005)

Metagenomic sequences from both Illumina sequencing of pooled fosmid libraries and direct 454 pyrosequencing of DNA isolated from an oil reservoir (Kotlar et al. 2011) were uploaded to MG-RAST. A series of metagenomic analysis tools in MG-RAST was applied to compare the functional and phylogenetic composition of the two sequence databases, and it was found that the two datasets were essentially similar in context of taxa distribution, with a high abundance of the domain *Archaea* (E value < 10^−5^), as well as their functional classifications (Fig. 1a, b).

### Identification of carbohydrate-degrading enzymes by sequence-based screening

The Illumina HiSeq sequencing of the fosmid library generated 40.1 Gbp of sequence reads (37.0 Gbp after trimming, with an average read length of 92 bp). Reads were assembled de novo, yielding 697,947 contigs. The ORFs predicted from these contigs were queried against the CAZy database using a local BLASTp search, leading to the discovery of 29,764 ORFs with significant BLAST hits (E value < 10^−5^). Based on the results of a local BLASTp against the CAZy database, we obtained 101 significant hits for cellulases, 21 hits for xylanases, 174 hits for amylases, 39 hits for proteases and 102 hits for esterases/lipases (Table 1). All cellulase, xylanase and amylase hits were described as members of the GH group.

**Table 1:** Number of positive hydrolase hits from functional and sequence-based screening of the oil reservoir metagenomic library

CAZyme	Functional screening		Sequence-based screening	
	No. of hits	Hit frequency (%)	No. of hits	Hit frequency (%)
Cellulase	6	0.052	101	0.88
Xylanase	2	0.017	21	0.18
Amylase	85	0.74	174	1.51
Protease	33	0.29	39	0.34
Esterase/lipase	9	0.078	102	0.89

In order to achieve greater contig lengths, Illumina HiSeq reads were sub-sampled from shotgun sequencing of Well II, as well as the corresponding plates from the fosmid library. Assembly of 90% of the shotgun reads yielded 4938 contigs with an average length of 2752 bp, which were further assembled with 20% of the fosmid library sequence reads, generating 655 contigs with average length of 6273 bp. Both contig sets were used for ORF prediction, followed by a local BLASTp search against the CAZy database resulting in a total of 1432 ORFs with significant BLASTp hits (E value < 10^−3^). Using this assembly approach, these hits yielded 13 significant hits for cellulases, 14 hits for xylanases, 34 hits for amylases, 35 hits for proteinases/peptidases and 29 hits for esterases/lipases (Table 1).

### Identification of carbohydrate-degrading enzymes by function-based screening

For each of the targeted CAZymes, we discovered a greater number of enzymes via sequence-based screening compared to function-based screening (Table 1). For cellulases, six validated clones were identified from functional screening (0.052% hit frequency), whereas 101 putative cellulase-encoding ORFs were identified from all sequence-based screening (0.88% hit frequency). Similar observations, with an expectedly higher hit frequency in sequence-based screening compared to function-based screening, were made for the five enzyme classes (Table 1), suggesting that many clones identified from sequence-based screening were not expressed or expressed in an inactive form in the *E. coli* heterologous host.

Among the different CAZy classes, we selected cellulases for further characterization due to their potential industrial application in the generation of cellulosic ethanol. All six clones that expressed a cellulase in initial functional screening were tested for their thermal stability. Three fosmid clones, named F1, F4, and F6 gave apparent halos on CMC agar assays after the clone supernatants had been incubated at elevated temperatures, while clones F2, F3, and F5 did not show any evidence of cellulase activity at higher temperature. Among the temperature-resistant clones, clone F1 had cellulolytic activity at high temperatures (60, 70 and 80 °C), whereas clones F4 and F6 did not show activity after a 80 °C incubation (data not shown). The cellulase activity expressed by F1 was observed to be the most thermostable and the most efficient at cellulose degradation based on this CMC substrate assay.

**Fig. 2:**
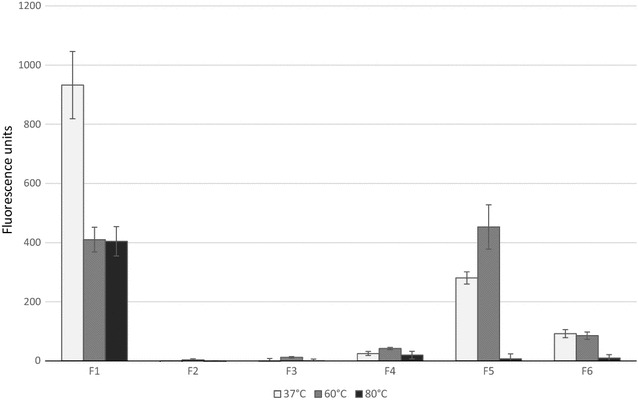
Quantitative MUC assay for six fosmid clones that were identified using a functional assay with the CMB substrate. The activity is reported as units of fluorescent signal intensity. Supernatants of the six clones were incubated at 37 °C (light grey), 60 °C (medium dark grey) or 80 °C (dark grey) to test the thermal stability of each cellulase. Error bars represent standard deviations from three independent measurements

The thermostability of each expressed cellulase was evaluated in a quantitative assay using the cellulase substrate MUC (Fig. 2). The cell lysate supernatant of each respective clone was heated at 37, 60, or 80 °C for 3 or 6 h, and then incubated with MUC. Clones F1, F4, F5, and F6 showed a strong fluorescent signal in the MUC assay. The activity of F1 using the MUC substrate was still the highest among all clones, even though this signal was reduced when the temperature was increased to 60 or 80 °C (Fig. 2). Clone F5 showed highest activity at 60 °C (Fig. 2), suggesting a temperature optimum around 60 °C. The cellulose-degrading activities of clones F4 and F6 were relatively weak as determined in the MUC assay, but since these data were not normalized per mg protein, this may also reflect lower expression of the cellulase rather than low enzymatic activity. The MUC activity of clone F6 was observed to gradually reduce with increasing temperature, whereas that of clone F4 remained stable at all three temperatures (Fig. 2).

### Sequence analysis of cellulase ORFs identified from both sequence-based and function-based screening

**Fig. 3:**
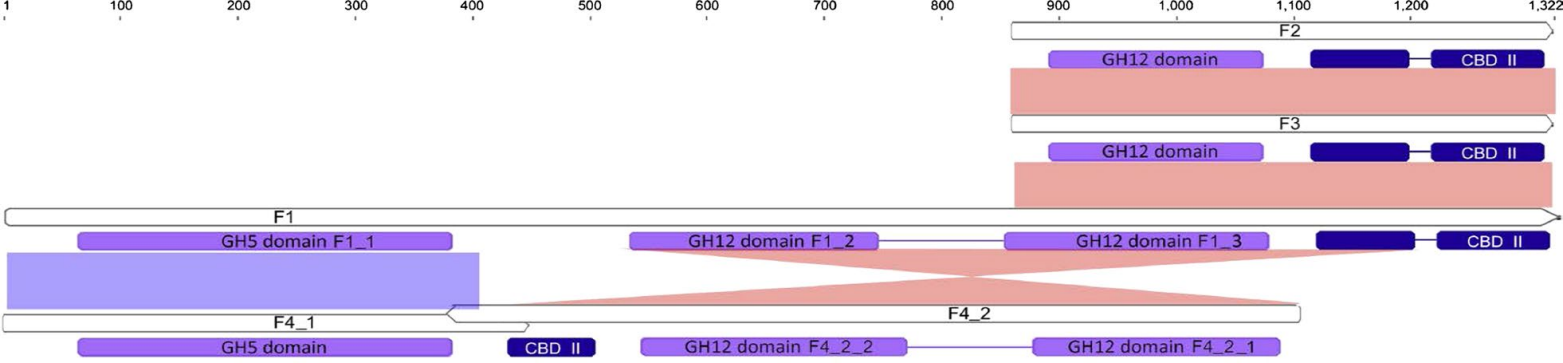
Module annotation of the cellulases F1, F2, F3, F4_1 and F4_2, as predicted by interproscan (Jones et al. 2014). Cellulase modules are labeled in purple, and CBM modules are labeled in blue. Identical regions between different cellulase sequences are presented in light blue and pink

**Fig. 4:**
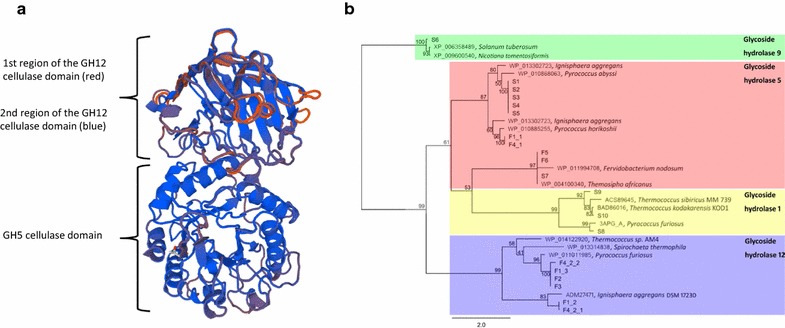
**a** 3D model of the cellulase F1 as predicted using the Swiss-Model server. The GH5-related cellulase module F1_1, residues 44 to 412, was modeled using 3axx.1.A (87.3% amino acid identity) as the template. The GH12-related cellulase modules were modeled using 3vgi.1.A. The first GH12-affiliated module (F1_2; residues 539 to 747, 35.8% amino acid identity) is depicted in red and the second GH12-affiliated module (F1_3; residues 830–1089, 82.7% amino acid identity) is depicted in blue. **b** Maximum likelihood phylogenetic analysis using amino acid sequences of cellulases identified in this study (in bold) and previously described cellulases derived from members of the domains *Eukaryota*, *Archaea* and *Bacteria*. 1000 iterations were conducted for bootstrap support, and bootstrap values are indicated at each node. Cellulases affiliated with GH9 are highlighted in green, GH12-affiliated sequences are highlighted in blue, GH5-affiliated sequences are highlighted in red and GH1-affiliated sequences are highlighted in yellow

The six cellulase expressing fosmid clones identified by functional screening were used to prepare sub-libraries with Nextera bar-codes and pooled for sequencing using Illumina MiSeq. The respective fosmid clones were separately analyzed, and a set of contigs was obtained for each clone, from which cellulase-encoding ORFs were detected (Additional file 1: Table S1). Interestingly, clone F1 indicated three predicted glycoside hydrolase (GH) modules that represent two different GH classes (Fig. 3). A module at the N-terminus, F1_1, exhibits 86.8% amino acid identity to the endocellulase of the archaeon *Pyrococcus horikoshii* (residues 67–385) and affiliates with the GH5 class. In contrast, the two modules at the C-terminus affiliate with the GH12 class, with module F1_2 exhibiting 59% amino acid identity to one glycoside hydrolase of the archaeon *Ignisphaera aggregans* DSM 17,230 (residues 537–749), and module F1_3 exhibiting 84.1% amino acid identity to the endo-1,4-β-glucanase of the archaeon *Pyrococcus furiosus* DSM 3638 (residues 856–1081) (Jones et al. 2014). In addition to the GH5 and GH12 modules, cellulase F1 is predicted to have a carbohydrate-binding module 2 (CBM2, residues 1227–1315) that may assist in binding cellulose substrates (Fig. 3). The predicted 3D structure of F1 was estimated using the Swiss-Model server using an endo-1,4-β-glucanase (3axx.1.A) and Endoglucanase A (3vgi.1.A) as templates to model the GH5 and GH12 modules, respectively (Fig. 4a) (Schwede et al. 2003). Since module F1_2 only has 35.8% amino acid identity to 3vgi.1.A, the weak homology of module F1_2 to known cellulase modules may preclude an accurate in silico model. For clones F2 and F3, the cellulase genes were predicted to have a GH12 module that is identical to the C-terminus of F1 (residues 866–1322) including the F1_3 module (Fig. 3, Additional file 1: Table S1). However, clones F2 and F3 lacked the GH5 module present in F1 (F1_1), and this could explain their apparent lack of thermostability. In the case of clone F4, there were two predicted overlapping cellulase ORFs (F4_1 and F4_2), both closely related to *Pyrococcus* spp. (Additional file 1: Table S1). Interestingly, F4_1 had one cellulase module of GH5 identical to that of F1_1, and F4_2 had two cellulase modules of GH12 (named F4_2_1 and F4_2_2) that were also identical to GH12-related modules of F1, but the direction of F4_2 was reversed compared to the region of F1 covering both F1_2 and F1_3 modules (Fig. 3, Additional file 1: Table S1). The overlapping region of F4_1 and F4_2 was actually the end of their C-terminus, which was 72 aa in length and distinct from that of the F1 GH12-related module (only 9.8% amino acid identity) (Fig. 3). Clone F5 contains a predicted cellulase that is identical to that of the cellulase predicted from clone F6, which has 99.7% amino acid identity to the endoglucanase of the bacterium *Thermosipho africanus* (Additional file 1: Table S1).

Despite a large number of cellulase ORFs discovered from the fosmid library (n = 101), there were only seven complete or nearly complete ORFs identified. This is likely due to the use of shorter Illumina HiSeq read lengths and the large number of fosmids in each pool, reducing the coverage per clone. Five of the ORFs identified from this search (named S1 through S5) had an identical DNA sequence with varied length and 70.6% amino acid identity to the endoglucanase of *Pyrococcus abyssi* GE5 (NP_126623) (Additional file 1: Table S1). Interestingly, the predicted cellulase ORF S6 had 85.3% amino acid identity to the endo-1,4-β-glucanase found from the tomato plant (*Solanum lycopersicum*). Since it is highly unlikely that a relative of that species is present in the oil reservoir and the coverage of the cellulase-encoding ORF was low, this perhaps represents a case of lateral transfer or contamination. All of these predicted ORFs except S6 were successfully amplified from pooled fosmid DNA. Only one sequence from a predicted cellulase ORF (S7) corresponded to the same sequence identified from a fosmid clone (F6) expressing a cellulase activity, and in this case the fosmid DNA was used as template for PCR.

The hybrid assembly of sequence reads derived from both the fosmid library and direct sequencing resulted in a larger number of complete cellulase-encoding ORFs (n = 13), compared to only using fosmid library reads (n = 7), and these were contained within larger contigs that included mostly intact ORFs. Putative cellulases discovered from the hybrid assembly include all cellulase ORFs discovered above except for S6. In addition to the cellulase ORFs described above, three additional cellulases (S8, S9, and S10) were identified from the hybrid assembly that are predicted to be β-glucosidases (Additional file 1: Table S1).

Since contigs derived from the hybrid assembly were much longer, all of the cellulase ORFs described above were mapped to these long contigs. This led to the discovery that two long contigs contain multiple cellulase ORFs. Contig_A (480,455 bp, the largest contig obtained from any of the assemblies) contained two cellulase ORFs, S8 and S10, as well as a complete rRNA operon (Additional file 1: Figure S1). The 16S rRNA gene of Contig_A has a top BLASTn hit to *Thermococcus celer* (98.5% nucleotide identity, M21529). Contig_B (279,442 bp) had three predicted cellulase ORFs, specifically S4, F1 and S9 (Additional file 1: Figure S1).

### Cellulase phylogenetic analysis

All cellulase ORFs discovered in this study and a selection of previously described cellulase gene sequences were used to construct a phylogenetic tree using PHYML to shed light on the evolutionary relationships of these enzymes (Fig. 4b). In the tree, the cellulase from S6 was distantly related to other identified cellulases, but clustered together with two known eukaryotic cellulases, presenting high similarity to that of *Nicotiana tabacum* and *Solanum tuberosum* that are affiliated with GH9. Several of the identified cellulases affiliated with GH5 and formed a monophyletic group with an archaeal cellulase from *Pyrococcus abyssi*. Cellulases with an identical amino acid sequence from clones F5 and F6, as well as from S7 were affiliated with GH5, which included bacterial cellulases from *Thermosipho africanus* and *Fervidobacterium nodosum*. The N-terminal module of clone F1 (F1_1) and cellulase of the ORF F4_1 (clone F4) were identical and both affiliated with the GH5 class and were in a clade together with an archaeal cellulase identified from *Pyrococcus horikoshii*. In contrast, the C-terminal modules from clone F1 (F1_2 and F1_3) and two modules from the ORF F4_2 (F4_2_1 and F4_2_2) were all clustered with archaeal cellulases of the GH12 class. Two cellulase modules, F1_2 and F4_2_1, were identical and affiliated with an archaeal cellulase identified from *Ignisphaera aggregans* DSM 17,230. The module F1_3 had an identical amino acid sequence with the module F1_2_2, as well as cellulases from clones F2 and F3, and the closest relative of them is an archaeal cellulase from *Pyrococcus furiosus*. In addition, three addition cellulases, putative β-glucosidases S8, S9 and S10, formed an independent clade with known archaeal cellulases from *Pyrococcus furiosus*, *Thermococcus sibiricus* MM 739, and *Thermococcus kodakarensis* KOD1, respectively, that affiliate with GH1. The phylogenetic analysis supports the monophyly of the bacterial and archaeal cellulases included in the analysis, and indicates that the thermostable cellulases identified in this study from *Archaea* represent novel clades, whereas the bacterial-derived cellulases were closely related to previously identified cellulases.

### Thermal stability of subcloned cellulases

**Fig. 5:**
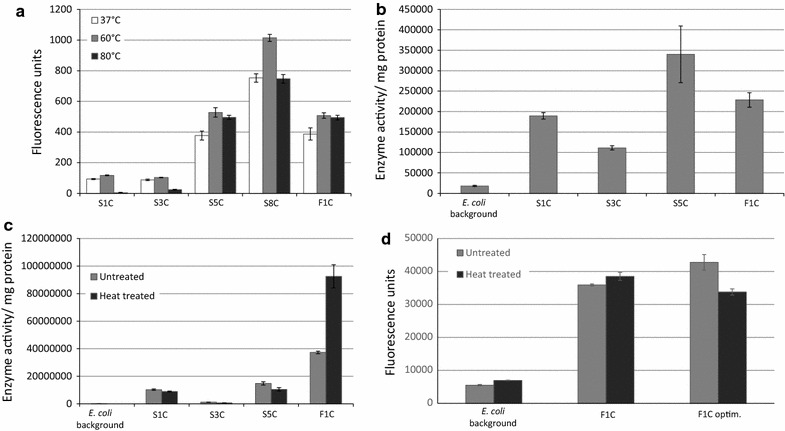
Activity analysis of cellulases and cellulase fractions measured by quantitative 4-MUC assay. **a** Analysis of supernatants of cell lysates from four subclones (S1C, S3C, S5C, S8C and F1C), in units of fluorescent signal intensity. The supernatants from each of the four subclones were incubated at 37 °C (light grey), 60 °C (medium dark grey) or 80 °C (dark grey), as indicated in figure, for 6 h to test the thermal stability of each respective cellulase. Values for a subclone with different superscripts (a, b, ab) were significantly different (*P* < 0.05) by one way ANOVA followed by Turkey multiple comparison. **b** Activity assay using crude cell extracts, heated at 65 °C for 20 min, with activity/mg protein plotted for extracts originating from *E. coli* expressing the four cellulase S1C, S3C, S5C, and F1C, in comparison to the *E. coli* negative control. **c** Activity assay of the Ni–NTA isolated protein, with activity/mg protein plotted for untreated (medium grey) as well as heat treated (dark grey; 65 °C, 20 min) samples. **d** Cellulase activity measured in crude cell extract from cells producing native enzyme F1 (F1C), codon optimized enzymes F1 (F1C optim.), as well as from cells not producing enzyme F1 (*E. coli* background). All samples were analysed with and without heat treatment (65 °C, 20 min). Error bars represent standard deviations from three independent measurements

Cellulase genes identified from both sequence-based and function-based screening were subcloned into the inducible expression Expresso-Rhamnose subcloning system (Lucigen, Middleton, WI). The resulting subclones were grown on CMC agar to assay for cellulase activity. Two of these subclones, S3C and S5C (the letter “C” denotes that these are subclones), showed apparent cellulolytic activity (data not shown). In contrast, using the MUC substrate, activity was detected from five subclones (Fig. 5a). The first four subclones were derived from sequence-based screening (S1C, S3C, S5C, and S8C), and the last one was from function-based screening (F1C). The sequences of S1, S3 and S5 are identical but have different lengths. S5 is 63 and 117 bp shorter than S1 and S3, respectively. Despite its shorter length compared to these other two subclones, S5C was observed to express a higher cellulolytic activity than S3C and S5C, and did not have a noticeable reduction in cellulase activity after being heated at 80 °C (Fig. 5a). In contrast, the cellulase activity expressed from subclones S1C and S3C was lower and decreased after heating at 80 °C (Fig. 5a). Interestingly, the subclone F1C expressed a significantly higher cellulase activity after heating at 60 and 80 °C compared to its activity at 37 °C (Fig. 5a). The subclone S8C had the highest activity against the MUC substrate among all five subclones and also had the highest activity after heating at 60 °C compared to the activity observed at 37 or 80 °C, indicating adaptation of this enzyme to a temperature close to 60 °C.

The thermal stability and activity of cellulase enzymes S1C, S3C, S5C, and F1C was analyzed in crude cell extracts, as well as isolated protein (after Ni–NTA affinity chromatography). Activities per mg protein in crude extracts containing these cellulases were significantly higher compared to the *E. coli* expression strain background (Fig. 5b), confirming heat stability, as well as cellulase activity of all four enzymes. The activity assay was repeated on the first elution fraction (eluted with 100 mM imidazol), with and without additional heat treatment (65 °C, 20 min) (Fig. 5c). The isolated cellulase from S3C did not show significant activity in the assay, suggesting poor yield in isolation in combination with a lower level of activity. Cellulases from S1C and S5C both showed activity as isolated proteins; however, a small decrease in activity was observed after heat incubation of the isolated proteins (Fig. 5c). For cellulase F1C, the measured activity of isolated protein was found to be remarkably higher compared to the other enzyme candidates. In addition, the cellulase activity expressed by F1C was very heat stable and the observed activity per mg protein increased notably after heat incubation of the isolated protein, indicating it is a very active and thermostable cellulase. Based on these results, further characterization therefore focused on enzyme F1.

Despite inducible gene expression for F1 cellulase production, no detectable protein band corresponding to the cellulase enzyme could be detected by SDS-PAGE (data not shown). Therefore, the corresponding synthetic gene was ordered as an *E. coli* codon-optimized variant (Genscript). Nevertheless, expression of the codon-optimized F1 gene still did not result in any detectable protein bands by SDS-PAGE and produced only very faint bands on a Western blot detecting the vector-encoded His-tag (data not shown). Even though soluble recombinant protein was apparently produced in small amounts, clear cellulase activity was confirmed for both the non-codon optimized, as well as the codon- optimized versions of the F1 cellulase, both in heated (65 °C, 20 min) and not heated crude cell extracts (Fig. 5d).

The F1 cellulase enzyme produced from the *E. coli* codon optimized gene variant was analyzed for substrate specificity and degradation product formation using several polysaccharide substrates. In order to be able to relate measured activity, the amount of total protein content in the heated crude cell extract was determined by Qubit measurement according to the manufacturer’s protocol to be 3.04 mg/ml. However, due to the inability to visualize the F1 cellulase by SDS-PAGE gel or by Western blot, it was unfortunately not possible to determine the concentration of the F1 enzyme in the heated or non-heated samples. Based on an estimated concentration of host proteins stained with Coomassie Brilliant Blue in SDS-PAGE analysis of the heated CCE sample, the F1 enzyme was estimated to represent not more than 5% of the total protein content, which would correspond to < 0.15 mg/ml.

**Fig. 6:**
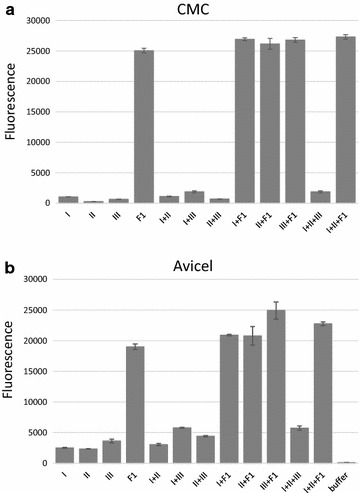
Activity of F1 (in heated, 65 °C, 20 min, crude cell extract) on **a** CMC and **b** Avicel as a substrate, detected as fluorescence by the Amplex Ultra Red based on H_2_O_2_ formed by glucose oxidase from enzyme reactions generating glucose. Enzyme I indicates endoglucanase (Sigma E2164), Enzyme II indicates cellobiohydrolase (Sigma E6412) and Enzyme III indicates beta-glucosidase (Sigma 49290). For assay using CMC, all enzymes were diluted 5x compared to the Avicel reaction in order to resolve results. Error bars represent standard deviations from three independent measurements

Enzyme F1 was found to be active on both CMC and Avicel substrates, both as a single enzyme and in combination with commercial available cellulases (Fig. 6). In contrast to the commercial enzymes endoglucanase (Sigma E2164), cellobiohydrolase (Sigma E6412) and β-glucosidase (Sigma 49290), F1 appeared to be active on CMC as a single enzyme, and resulted in notably higher fluorescence (H_2_O_2_ detected, and hence glucose produced) than an enzyme cocktail of enzymes endoglucanase, cellobiohydrolase and β-glucosidase. Similar to the results using CMC as a substrate, also for the microcrystalline cellulose substrate Avicel, F1 proved to be functional as a single enzyme, which was not observed for the commercially available enzymes used. It also showed higher activity compared to the complete cocktail of the three reference enzymes. The amount of F1 enzyme used in the substrate specificity reaction assays was estimated to be (maximum) 0.003 mg/ml (20 µl of 0.15 mg/ml stock solution) or (maximum) 0.0006 mg/ml in CMC assay (5× diluted enzyme used). This is likely an overestimation of the actual F1 enzyme amount used; however, this was much lower than the amounts of commercial enzymes applied, such as 0.021 mg/ml for endoglucanase (Sigma E2164), 0.039 mg/ml for cellobiohydrolase (Sigma E6412) and 0.011 mg/ml for β-glucosidase (Sigma 49290).

**Fig. 7:**
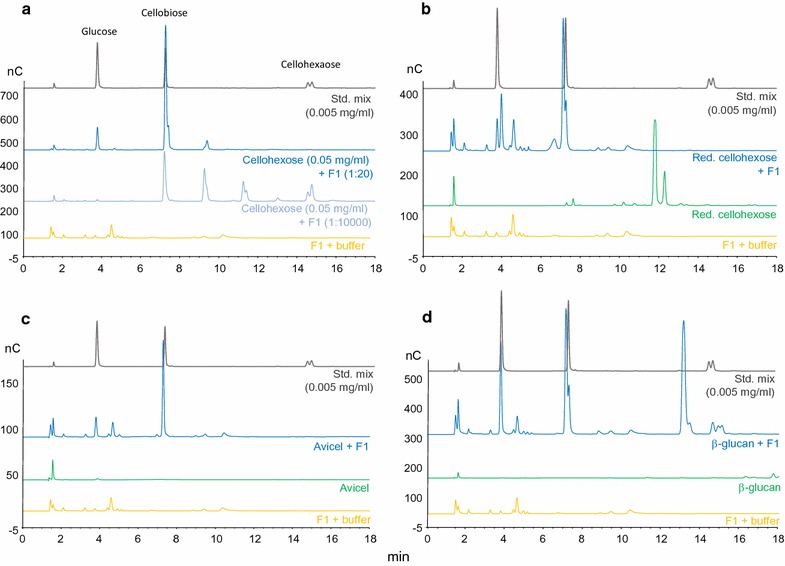
Results from ICS analysis of degradation products using enzyme F1 on different substrates. In all cases, reactions were performed in a 1 ml volume containing 50 µl F1 enzyme, i.e. 1:20. A standard mixture (std mix) applied gives peaks for cellohexaose, cellobiose and glucose, indicated in panel: **a** Cellohexaose used as reaction substrates, with 50 µl (1:20) or 0.1 µl (1:10,000) F1 enzyme. Intermediate product peaks detected in low enzyme amount reaction, from right to left pentamer, tetramer, trimer followed by cellobiose and glucose. **b** Reduced cellohexaose used as reaction substrate. **c** Avicel used as reaction substrate (reaction products diluted 1:10 prior to analysis by ICS). **d** β-glucan used as reaction substrate. For the full chromatogram of β-glucan w/wo F1 added, please refer to Additional file 1: Figure S2

Further, the F1 enzyme–substrate specificity was assayed using ICS and different substrates, which revealed that this enzyme degrades both cellohexaose and Avicel (to a lesser degree) into cellobiose and to some extent glucose (Fig. 7). Levels of glucose produced were relatively low but clearly detectable. By decreasing the amount of enzyme used in the cellohexaose degrading reaction, several intermediate products became detectable, i.e. penta-, tetra-, and triose, in addition to cellobiose and glucose (Fig. 7a). Degradation of NaBH_4_-reduced cellohexaose resulted in the same degradation pattern as the non-reduced substrate, indicating that the redox state of this substrate did not significantly affect F1 enzymatic activity (Fig. 7b). It was observed that F1 did not have any activity on the substrates cellobiose, xyloglucan, laminarihexose, maltoheptaose, maltohexaose or chitin (data not shown). The activity of enzyme F1 on Avicel was confirmed by ICS, leading to the production of cellobiose and glucose (Fig. 7c). In addition, it was determined that F1 is active on beta-glucan substrate, where it again generated cellobiose and glucose as reaction products (Fig. 7d). Incubation of pre-hydrolysed β-glucan with enzyme F1 also resulted in full degradation of the substrate (data not shown).

## Discussion

The results of the present study confirm our earlier analyses that the processed oil reservoir sample was dominated by members of the domain *Archaea*, phylum *Euryarchaeota* (Kotlar et al. 2011; Lewin et al. 2014). The results obtained from direct 454 pyrosequencing and from the Illumina sequencing of the fosmid library were highly comparable in terms of phylogenetic and functional composition (Fig. 1a, b). The alpha-diversity of the shotgun sequences database as determined based on Shannon’s Diversity Index was 42.08 species, which is relatively low compared to non-extreme environments. Despite of a few exceptions, the overall high consistency of the sequencing results indicates that overall biases are relatively low. Given the observation that the *Archaea* are dominant in this environment and that most of the enzymes obtained in this study were predicted to be derived from taxa affiliated with the *Euryarchaeota*, we conclude that our sampling of these oil reservoir microbial assemblages has been inclusive of much of the extant phylogenetic and functional diversity. The large number of unassigned sequences from both direct and library sequencing indicates that even though this is an extreme habitat with limited phylogenetic breadth, there is a considerable amount of previously unknown metagenomic diversity in the sampled environment.

Inferences of the functional capacity of these oil reservoir microorganisms gleaned from MG-RAST output indicated that carbohydrate-degrading enzymes are frequently encoded within the archaeal and bacterial genomes (Fig. 1b). However, crude oil consists primarily of hydrocarbons of various molecular weights, and one would predict that only small amounts of carbohydrates such as cellulose, starch and xylan, if any, exist in deep sub-surface oil reservoirs. While the concentrations of these carbohydrates were not determined from oil samples, the assumption is that these carbohydrates are present in limited amounts and are probably from remnant biomass. Alternative functions of polysaccharide hydrolases in organisms from oil reservoir samples may be in the metabolism of storage polysaccharides or extracellular polysaccharides (EPS) formed by many organisms, including hyperthermophilic *Archaea* (Rinker and Kelly 1996). In addition, we observed that some of the CAZymes discovered from sequence- or function-based screening were redundant, indicating that the methods we used in this study had sufficiently exhausted much of the enzymatic diversity present in these samples and that there is a limited overall diversity of CAZymes in this hyperthermal habitat. Surprisingly, a cellulase gene S6 was discovered to have homology with a cellulase from *Solanum lycopersicum*, the garden tomato. The contig from which S6 is derived is very short with very low coverage for its assembly, and was only assembled from reads derived from the cloned fosmid library. It may indicate a potential gene transfer event or a sample contamination during DNA extraction. The lack of additional sequences linked to this predicted cellulase gene precludes a more precise conclusion.

Five subclones were generated that were observed to have significant cellulase activity in the quantitative MUC assay. Subclones F1C, S5C and S8C showed good thermal stability in the MUC assay at both 60 and 80 °C, and were therefore the best prospects for thermostable cellulases for potential future applications. The S8 ORF was located on contig_A, which was 480,455 bp and the longest among all assembled contigs (Additional file 1: Figure S1). Fortunately, this contig included an intact rRNA operon, with the 16S rRNA gene from contig_A having 98.5% identity with the 16S rRNA gene from *Thermococcus celer*. Among the predicted ORFs from contig_A, 85% of these ORFs (446 out of 523) had a top BLASTp hit to a gene product from the genus *Thermococcus.* This is very robust evidence that contig_A and the cellulose-encoding genes present on this contig were derived from a *Thermococcus* species. Contig_B (279,442 bp) contained sequences of the S4 and F1 ORFs (Additional file 1: Figure S1). The S5 cellulase is identical to, but 42 aa shorter than the S4 cellulase, and it is possibly an incomplete ORF (belonging to part of S4) assembled from a short contig. Interestingly, although having a truncated ORF compared to S4, the subcloned S5 cellulase was observed to have significant cellulase activity against the MUC substrate whereas the S4 cellulase was negative for activity against MUC. No 16S rRNA gene was identified on contig_B, but 88% of the top BLASTp hits (274 out of 312) from predicted ORFs on contig_B also had significant similarities to the genus *Thermococcus*. Furthermore, an analysis of codon usage from clone F1 sequences also supports an origin from *Thermococcus* spp. rather than from *Pyrococcus* spp. (data not shown). Taken together, these results suggest that both of the highly active and thermostable cellulases identified from this study were derived from thermophilic *Archaea* within the genus *Thermococcus* that was predicted to be the dominant genus in the environment by MG-RAST, and that expression of these archaeal cellulases was possible (at least in some cases) from native archaeal promoters expressed in an *E. coli* heterologous host.

A phylogenetic analysis of the cellulases obtained from this study supports the affiliation of these cellulases with cellulases previously obtained from all three domains of life. Our data indicates that the majority of the cloned cellulases, and other CAZymes, are affiliated with *Archaea* and *Bacteria*, particularly with taxa affiliated with the phylum *Euryarchaeota*. Interestingly, all of the bacteria-derived cellulases are affiliated with the GH5 category, with many archaeal-derived cellulases also affiliated with this clade (highlighted in red, Fig. 4b). GH5 is one of the largest of the CAZy GH families and contains cellulases widely derived from *Bacteria*, *Archaea* and even *Eukaryota*. In contrast, other archaeal cellulases were classified within the category of GH1, GH5 and GH12. Archaeal cellulases affiliated with GH12 were all identified from function-based screening (highlighted in blue, Fig. 4b) and were subsequently discovered independently from the hybrid assembly of fosmid library and direct sequencing reads. Conversely, the three archaeal cellulases affiliated with GH1 are putative β-glucosidases, which were only identified from sequence-based homology searches (highlighted in blue, Fig. 4b). Therefore, there were examples of bias in the discovery of cellulases depending on whether a function-based or sequence-based screening method was used. Presumably, the lack of identification of some cellulases by sequence analysis was a result of low sequencing depth of the fosmid library reads for particular clones in the pooled library format. The inability to identify some cellulases in function-based screening was anticipated and may be explained by a potential lack of expression of the enzymes from their native promoters in the *E. coli* expression host, an inability to be translated due to differing codon usage, or an inability to be secreted and/or active under the conditions used for functional screening. The observation that we obtained distinct cellulase types using function- and sequence-based screening methods highlights the potential biases associated with metagenome mining methods and supports the use of multiple approaches to identify novel natural products from environmental metagenomes.

The cellulase F1 was encoded by the longest ORF among all of the identified cellulases, and its subclone gave rise to the highest cellulase activity using MUC as a substrate. Interestingly, F1 is predicted to have three distinct cellulase modules that affiliate with glycoside hydrolases within the GH5 and GH12 classes. Homologies of these three modules against known cellulases are low, in particular F1_2 with only 59% amino acid identity to its top BLASTp hit. From the phylogenetic analysis, it is apparent that the three modules have distinct lineages (Fig. 4b). Although the F1_2 and F1_3 modules are affiliated with the same class GH12, they are affiliated with two different independent clades (Fig. 4b). Furthermore, it is likely that the archaeal cellulase F1 evolved from fusion of two cellulases from distinct families, potentially resulting in its strong cellulase activity and thermal stability. However, to our knowledge no known cellulase containing these three modules has been previously identified. Also, CBM2 modules mainly exist in bacterial enzymes, and only six of the modules (out of 1953 described to date) were reported to be of archaeal origin in the CAZy database (Lombard et al. 2013). These data suggest that the fusion of these two cellulolytic modules resulted in a novel protein structure with enhanced thermal stability and distinct activity profile.

From studies on substrate specificity of enzyme F1 it was found that F1 is active on both CMC and Avicel as a single enzyme, in contrast to the commercially available enzymes endoglucanase (Sigma E2164), cellobiohydrolase (Sigma E6412) and β-glucosidase (Sigma 49290), and can produce cellobiose and to some extent glucose. Remarkably, the F1 enzyme was used at lower amounts than the commercial enzymes and still showed comparatively high activities, indicating that F1 is a highly active enzyme on polymeric cellulosic material. Chromatography-based methods were applied to identify reaction products of the F1 enzyme on Avicel, as well as get further insight into substrate specificity and processivity of the enzyme. From this, it is clear that F1 acts on crystalline cellulosic material and produces preferably cellobiose, but surprisingly also to some extent generates glucose as a by-product. The enzyme exhibits exo-activity on cellulose polymers as no soluble oligomers larger than cellobiose can be obtained from Avicel (in particular no cellotriose), while cellotriose is detected from processing cellohexaose as a substrate. This indicates that the F1 enzyme has activity only on substrates larger than three glucose units. In particular, the detection of significant amounts of glucose (in addition to the main degradation product cellobiose) is surprising and represents a potentially very valuable feature of this enzyme with respect to the degradation of (ligno)cellulosic biomass. In addition, using ICS it was determined that the F1 enzyme is active on cellohexaose and beta-glucan, as well as hydrolysed beta-glucan (Mw = 89 kDa), generating both cellobiose and some glucose, and that this enzyme generates a similar degradation pattern when acting on reduced cellohexaose as a substrate. The enzyme did not show any activity on cellobiose, xyloglucan, laminarihexaose, chitin, maltoheptaose or maltohexaose. The high activity of the F1 enzyme on microcrystalline cellulose with degradation to cellobiose, as well as to some extent glucose, as well as its high thermostability indicate its potential value as an industrial cellulolytic enzyme. Future studies will target the development of optimized heterologous production systems, in particular for the F1 enzyme in order to achieve yields detectable on SDS-PAGE. Subsequently, this will enable investigations of the three-dimensional protein conformation for the thermal stable cellulases, their kinetic parameters and the structure–function relationships important for cellulase activity against multiple substrates and stability under environmental extremes.

In conclusion, this study revealed that an oil reservoir microbial assemblage harbored novel metagenomic diversity and could be mined for thermostable cellulases and other CAZymes using a combination of function- and sequence-based methods, demonstrating the strength of hybrid screening approaches. The results of this study have provided novel thermostable archaeal cellulases that are stable up to at least 80 °C. One of the cellulase candidates, enzyme F1, was demonstrated to be a multi-module, thermostable archaeal enzyme with high activity on different cellulose substrates, producing cellobiose and glucose in a single enzyme reaction.

## Additional file

**Additional file 1.** Additional table and figures.

## Abbreviations

ORF: open reading frame
BLAST: basic local alignment search tool
CAZy: carbohydrate-active enzymes
GH: glycoside hydrolase
SSF: simultaneous saccharification and fermentation
SHF: separated hydrolysis and fermentation
WGA: whole genome amplification
CMC: carboxymethylcellulose
MUC: 4-methylumbelliferyl-β-d-cellobioside
CCE: crude cell extract
DTT: dithiothritol
HRP: horse radish peroxidase
MWCO: molecular weight cutoff
CBM2: carbohydrate-binding module 2
EPS: extracellular polysaccharides
