# Supplementary material for: Novel archaeal thermostable cellulases from an oil reservoir metagenome
Source: AMB Express. 2017 Sep 29;7:183. doi: 10.1186/s13568-017-0485-z (PMC5622026; doi:10.1186/s13568-017-0485-z)
Supplement: Supplementary file 1 — Additional file 1. Additional table and figures. [file 13568_2017_485_MOESM1_ESM.pdf]

Supplementary Material

Novel Archaeal Thermostable Cellulases from an Oil Reservoir Metagenome

Anna Lewin[1†], Jinglie Zhou[2†], Vu Thuy Trang Pham[2,3], Tone Haugen[1], Mohamed El Zeiny[2], Olav Aarstad[4], Wolfgang Liebl[3], Alexander Wentzel[1*] and Mark R. Liles[2*]

[1]SINTEF Materials and Chemistry, Department of Biotechnology and Nanomedicine, Trondheim, Norway. [2]Department of Biological Sciences, Auburn University, Auburn, AL, USA. [3]Department of Microbiology, Technical University of Munich, Freising-Weihenstephan, Germany. [4]Norwegian University of Science and Technology (NTNU), Trondheim, Norway.

†These authors contributed equally to this manuscript

*Corresponding authors:
Mark R. Liles
Address: 101 Life Sciences Building, 120 W. Samford Avenue, Auburn, AL 36849
Phone: +1 (334) 844-1656 (office)
Email: lilesma@auburn.edu
Alexander Wentzel
Address: Richard Birkelands vei 3B, 7465 Trondheim, Norway
Phone: +47 93200776
Email: alexander.wentzel@sintef.no

Running title: Novel archaeal cellulases from oil metagenome

Table S1. Origin of cellulase enzyme harbouring clones and their annotation.

**Cellulase candidate**	**Module**	**Taxa annotation**	**Similarity**	**Annoated function**	**Annotated class**
F1	F1_1	*Pyrococcus horikoshii*	86.8%	endocellulase	GH5
	F1_2	*Ignisphaera aggregans* DSM 17230	59%	endocellulase	GH12
	F1_3	*Pyrococcus furiosus* DSM 3638	84.1%	endo-1,4-beta-glucanase	GH12
	F1_4			carbohydrate-binding module 2	CBM2
F2	F2_1	*Pyrococcus furiosus* DSM 3638	84.1%	endo-1,4-beta-glucanase	GH12
F3	F3_1	*Pyrococcus furiosus* DSM 3638	84.1%	endo-1,4-beta-glucanase	GH12
F4	F4_1	*Pyrococcus furiosus*	78.8%	endo-1,4-beta-glucanase	GH5
	F4_2_1	*Pyrococcus horikoshii*	79.6%	endocellulase	GH12
	F4_2_2	*Pyrococcus horikoshii*	79.6%	endocellulase	GH12
F5	F5_1	*Thermosipho africanus*	99.7%	endoglucanase	GH5
F6	F6_1	*Thermosipho africanus*	99.7%	endoglucanase	GH5
S1	S1_1	*Pyrococcus abyssi* GE5 (NP_126623)	70.6%	endoglucanase	GH5
S2	S2_1	*Pyrococcus abyssi* GE5 (NP_126623)	70.6%	endoglucanase	GH5
S3	S3_1	*Pyrococcus abyssi* GE5 (NP_126623)	70.6%	endoglucanase	GH5
S4	S4_1	*Pyrococcus abyssi* GE5 (NP_126623)	70.6%	endoglucanase	GH5
S5	S5_1	*Pyrococcus abyssi* GE5 (NP_126623)	70.6%	endoglucanase	GH5
S6	S6_1	*Solanum lycopersicum*	85.3%	endo-1,4-beta-glucanase	GH9
S7	S7_1	*Thermosipho africanus*	99.7%	endoglucanase	GH5
S8	S8_1	*Pyrococcus furiosus* (WP_011011185)	77.5%	beta-glucosidases	GH1
S9	S9_1	*Thermococcus kodakarensis* (WP_048053751)	73.1%	beta-glucosidases	GH1
S10	S10_1	*Thermococcus kodakarensis*	96.1%	beta-glucosidases	GH1

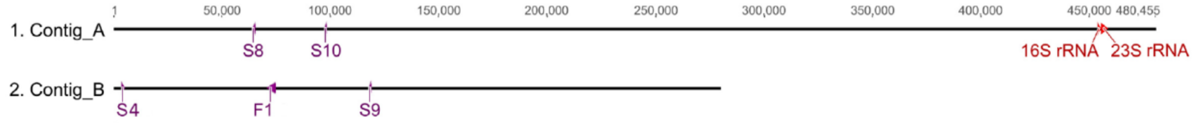

Figure S1. Sequence annoration for contig_A and contig_B, indicating the predicted cellulase ORFs in purple. The rRNA operon, including 16S rRNA and 23rRNA genes on contig_A, annotated in red, was predicted using RNAmmer v. 1.2.

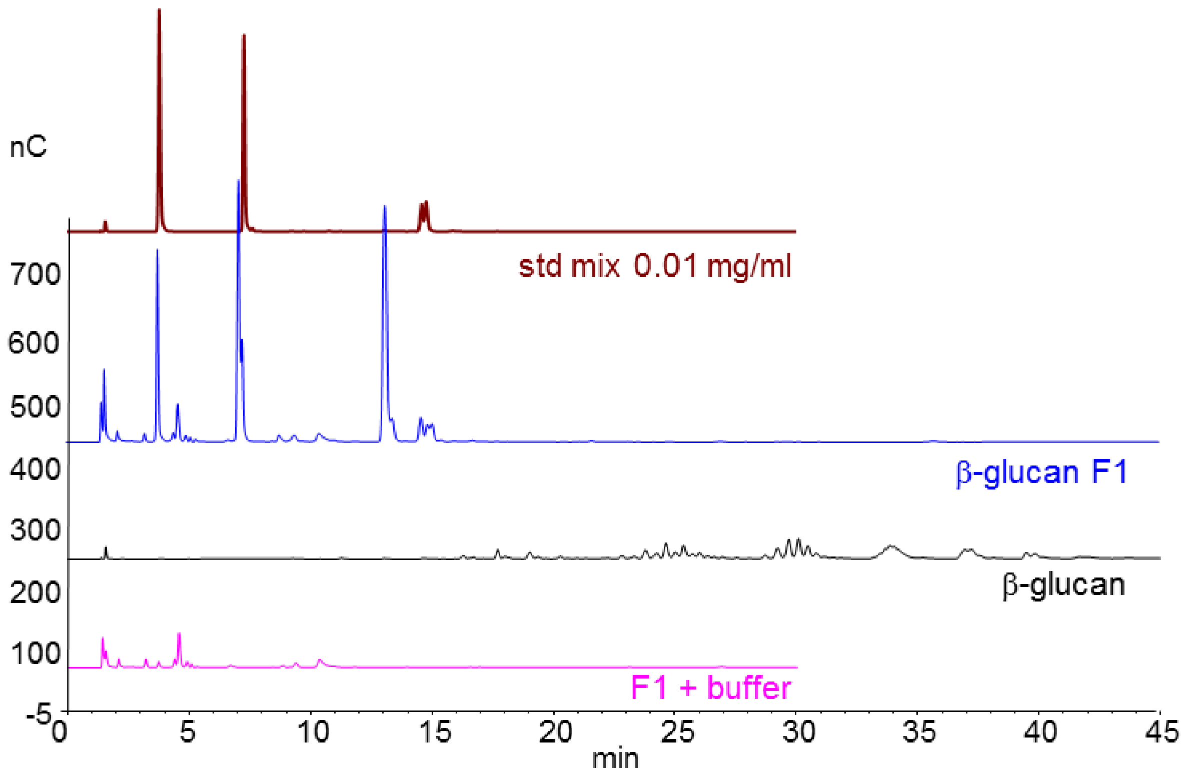

Figure S2. Results from ICS analysis of degradation products using enzyme F1 on β-glucan. Reaction was performed in a 1 ml volume containing 50 µl F1 enzyme, i.e. 1:20. A standard mixture (std mix) applied gave peaks for cellohexaose, cellobiose and glucose, indicated. The figure shows the full chromatogram from β-glucandegradation, to supplement Figure 7.
